# Gamification and Game-Based Strategies for Dermatology Education: Narrative Review

**DOI:** 10.2196/30325

**Published:** 2021-08-30

**Authors:** Mindy D Szeto, Daniel Strock, Jarett Anderson, Torunn E Sivesind, Victoria M Vorwald, Hope R Rietcheck, Gil S Weintraub, Robert P Dellavalle

**Affiliations:** 1 Department of Dermatology University of Colorado Anschutz Medical Campus Aurora, CO United States; 2 School of Medicine Eastern Virginia Medical School Norfolk, VA United States; 3 Arizona College of Osteopathic Medicine Glendale, AZ United States; 4 School of Medicine University of Colorado Anschutz Medical Campus Aurora, CO United States

**Keywords:** games, game-playing, gamification, serious games, simulations, education, medical education, dermatology education, patient education, review

## Abstract

**Background:**

Game-based approaches, or gamification, are popular learning strategies in medical education for health care providers and patients alike. Gamification has taken the form of serious educational games and simulations to enable learners to rehearse skills and knowledge in a safe environment. Dermatology learners in particular may benefit from gamification methods, given the visual and procedural nature of the field.

**Objective:**

This narrative review surveys current applications of gamification within general medical training, in the education of dermatology students, and in dermatology patient outreach.

**Methods:**

A literature search was performed using PubMed, Google Scholar, and ResearchGate to access and review relevant medical education- and dermatology-related gamification studies published in peer-reviewed journals. Two independent researchers with education and experience in dermatology screened publications to select studies featuring a diversity of gamification approaches and study subjects for in-depth examination.

**Results:**

A total of 6 general medical education–related and 7 dermatology-specific gamification studies were selected. Gamification generally increased motivation and engagement, improved reinforcement of learning objectives, and contributed to more enjoyable and positive educational experiences compared to traditional modes of instruction. Enhancing examination scores, building confidence, and developing stronger team dynamics were additional benefits for medical trainees. Despite the abundance of gamification studies in general medical education, comparatively few instances were specific to dermatology learning, although large organizations such as the American Academy of Dermatology have begun to implement these strategies nationally. Gamification may also a provide promising alternative means of diversifying patient education and outreach methods, especially for self-identification of malignant melanoma.

**Conclusions:**

Serious games and simulations in general medical education have successfully increased learner motivation, enjoyment, and performance. In limited preliminary studies, gamified approaches to dermatology-specific medical education enhanced diagnostic accuracy and interest in the field. Game-based interventions in patient-focused educational pilot studies surrounding melanoma detection demonstrated similar efficacy and knowledge benefits. However, small study participant numbers and large variability in outcome measures may indicate decreased generalizability of findings regarding the current impact of gamification approaches, and further investigation in this area is warranted. Additionally, some relevant studies may have been omitted by the simplified literature search strategy of this narrative review. This could be expanded upon in a secondary systematic review of gamified educational platforms.

## Introduction

Game-based approaches, or gamification, are becoming increasingly popular in health care education. These novel and innovative strategies use game design elements such as the concept of a player or players, rules, conflicts, and predetermined goals in an artificial setting [1,2]. Previous literature has formally defined gamification as the application of game characteristics and benefits to real-world processes or problems [3]. The concept of gamification has been separated from that of *serious games*, which are complete games for specifically educational, nonentertainment purposes [3,4]. Related to but outside the strict definition of serious games are *simulations*, defined as virtual or “concentrated” realities offering opportunities for learners to rehearse skills and knowledge [1]. Because these delineations have been the subject of some debate [3], for the purposes of this review, we will broadly use gamification to refer to both serious games along with other applications leveraging game and simulation elements for a utilitarian purpose.

**Figure 1:**
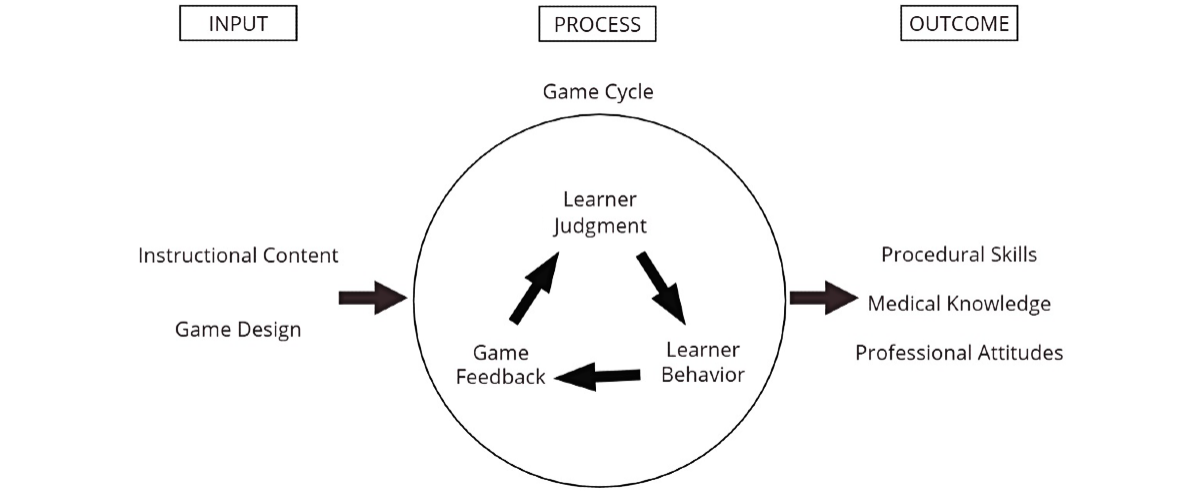
Input-process-outcome model of game-based learning [12] adapted for medical education.

Generally, gamification provides a structured, safe, and low-risk environment for learners to build skills and confidence without real-world consequences [5,6]. It can help students to engage in a particular activity, think critically about both their plan and outcome, and then apply important insights gained from their analyses to improve and learn. Emerging digital technologies have transformed how gamification strategies can be used in education. With the dramatic rise of digital gaming as a widespread pastime, incoming students and the general public are more accustomed to gaming in their daily lives than ever before [3,7,8], which presents an ideal opportunity. Studies have shown that students prefer gamification over traditional educational curricula [9,10], especially if they enjoy diverse and alternative learning styles such as primarily visual, auditory, or kinesthetic methods [11]. A general model [12] of how game-based learning achieves desired outcomes in medical education, in which the game cycle is an iterative process of learner judgment, behavior, and feedback, is demonstrated in Figure 1.

Many gamification formats in medical education have been presented including simulations, virtual/alternative reality environments, and social and cooperative gaming [1]. Moreover, the practice-focused nature of health care allows gamification to provide a cost-effective method of optimizing procedural skills such as thoracentesis [13]. Patients also stand to benefit greatly from gamification [14]. Compelling aspects of games such as rewards, competition, self-expression, and social interaction have been extracted to encourage healthy habits and track fitness goals in many popular programs and mobile apps. Game-based challenges can also benefit patients by driving frequent patient engagement, increasing education through quizzes and daily tips, and by motivating difficult long-term behaviors such as medication adherence [15].

Dermatology, as a highly visual and procedural field, is well positioned to take advantage of the learning methods employed in many game-based approaches. This narrative review therefore aims to survey gamification in medical training as well as to explore how the current knowledge base of gamification applications can be expanded in dermatology education for both providers and patients alike through further evidence-based examination of game-based learning.

## Methods

A survey of peer-reviewed scientific literature was performed throughout January and February of 2021, with the purpose of identifying studies for a narrative review of gamification in health care education and dermatology. Key search terms included *gamification*, *games*, *game-playing*, *serious games*, and *simulation games*, in combination with terms such as *healthcare*, *healthcare education*, *medical education*, *dermatology*, and *dermatology education*. PubMed electronic database searches were supplemented by Google Scholar and ResearchGate to retrieve the full English-language text of each article and remove duplicate search results. The articles included studies from multiple academic institutions worldwide, and they included randomized controlled trials, cohort studies, and case studies. The participants in the studies encompassed medical students, medical residents, and members of the general public. To provide a broad overview of gamification and game-based interventions, both serious games and simulations were surveyed. Two independent researchers with education and experience in dermatology performed separate screenings of the article titles and abstracts, ultimately selecting 6 general medical education–related studies and 7 dermatology-related studies featuring a diversity of gamification approaches and study subjects for in-depth discussion in this narrative review. Additional consultation with board-certified dermatologists involved in medical education led to the inclusion of two recent *Jeopardy* game show–like events tailored toward dermatology residents at national conferences. Table 1 provides a summary of the studies and various gamification approaches examined.

**Table 1:** Summary of the gamification approaches and studies surveyed.

Game	Classification	Target audience	Participants studied, n	Topic
Kaizen [16]	Serious game	Medical residents	94	Clinical knowledge
SIU^a^ uRCADE [17]	Serious game	Clinicians and medical students	Unknown	Bladder cancer and renal cell carcinoma
Stanford 360 VR^b^ [18]	Simulation	Clinicians and medical students	207	Mass pediatric casualty incident
Recreational escape room [19]	Blended	Emergency medicine residents	10	Teamwork
Team-based competition [20]	Serious game	General surgery residents	Approximately 50	Medical knowledge, patient care, professionalism, interpersonal and communication skills, scholarship
Video games and *Jeopardy*-style contest [21]	Blended	Cardiothoracic surgery residents	43	Procedural skills and surgical knowledge
Kahoot! [22]	Serious game	Medical students	51	Medical knowledge, histopathology, tumor identification
Skinquizition [23]	Serious game	Medical students	384	Skin diseases and applied dermatology knowledge
American Academy of Dermatology Annual Meeting Resident Jeopardy [24]	Serious game	Dermatology residents	Unknown	Dermatological conditions
DermPath Bowl [25]	Serious game	Dermatology residents	48 residency programs	Dermatopathology
i-DERMIFY [26]	Serious game	Medical students	28	Dermatological conditions
Stud2yBuddy [27]	Serious game	Medical students	65	Dermatological conditions
“Zombie apocalypse” escape room [28]	Blended	Medical students	16	Clinical knowledge, attitudes toward the field of dermatology
Mountain View High School Survey [29]	Serious game	Patients (adolescents)	271	Malignant melanoma identification
Tapamole [30]	Serious game	Patients	60	Malignant melanoma identification

^a^SIU: Société Internationale d’Urologie.

^b^VR: virtual reality.

## Results

### Gamification in General Medical Education

Gamification has been employed by medical schools and training programs to engage learners outside the traditional text-based or didactic setting. The effectiveness of gamification in general medical education has been the subject of several studies, many of which examine the impact of game-based learning on objectives such as knowledge, skills, professional attitudes, or satisfaction. Although we retrieved several hundred articles in our initial literature search, the vast majority discussed gamification approaches in general medical education and were not specific to dermatology learning. We discuss a selection of applications of gamification in general medical education below.

One example of a serious game in general medical education is Kaizen, which tested clinical knowledge of medical residents in several university departments using a web-based multiple-choice quiz [16]. Achievement badges were earned for answering questions daily or answering multiple questions correctly in a row. To align with evolving technological experiences of incoming learners, future Kaizen developments may include Android/iPhone mobile apps and “boss battle” features or leaderboards for team challenges [31]. Indeed, many organizations have introduced serious educational gaming applications to engage medical learners. The Société Internationale d’Urologie (SIU, the International Society for Urology) recently launched web-based applications focusing on bladder cancer and renal cell carcinoma through their official education gaming arcade (SIU uRCADE), and they experienced overwhelming demand [17]. Participants could even earn approved Continuing Medical Education (CME) credits by completing uRCADE games, highlighting their effectiveness and utility.

Virtual reality and simulation games have proved to be very popular among medical learners of all levels. Stanford University recently used the existing 360 Virtual Reality platform, which is already used in entertainment, military training, and pilot training, to create a training module for mass casualty incident training [18]. Actors created realistic scenes of a care point involving 150 potential pediatric casualties, with the immersive virtual reality story allowing users to select triage categories and decide on interventions. The user would then see the intervention as it took place and would learn of the child’s outcome, earning points and achievement levels based on their responses. Attending physicians, residents, and students all reported the virtual reality simulation to be enjoyable and engaging, more so than traditional mannequin-based simulations. They also reported feeling more prepared to respond to a pediatric mass casualty incident after completing the exercise.

Blended gamified approaches and applications of gamification to traditional education have likewise been favorably received. For instance, an “escape room” game in which groups solved puzzles and completed tasks within a time limit boosted team-building and multitasking skills for emergency medicine residents. These games may be effective approaches to fulfill difficult-to-teach core Accreditation Council for Graduate Medical Education competencies, such as teamwork [19]. Incorporating game-based elements into existing curricula has also been fruitful. Team-based competitions converting surgical resident performance into game points with leaderboards and *Jeopardy*-style game shows were found to increase resident satisfaction, training scores, and board examination pass rates. The competitive nature of the trainees may be a contributing factor to the success of the gamification interventions [20,21]. Corroborating this, leaderboards have been suggested to be the most important motivator of game participation among medical residents, while nondaily play worsened attrition [32]. Integration of competitive gaming features with public platforms such as Twitter was found to increase professional social media presence and trainee engagement with their specialty [33]. Video games were also seen to effectively supplement surgical skills education in laparoscopy and robotic surgery [34]. Clearly, synergistic combinations of serious games, simulations, and supplementing traditional education with games and game-based elements may be enjoyable and effective for participants.

### Gamification in the Education of Dermatology Students

While gamification has been shown to have a positive impact on general medical education, we also sought to examine whether this held true in dermatology-specific game-based learning. The visual and procedural nature of dermatology would be expected to align well with the gamified educational methods discussed previously. As with gamification in general medical education, every dermatology-focused study reported beneficial effects of gamification on learning outcomes or student satisfaction, although it appears that only 7 dermatology-specific games are reported in the literature overall. Thus, further research and game development is needed in this area to fully elucidate the impact and future dermatology-specific utility of gamification.

One instance of adapting games previously used in general medical education for dermatology students was seen in Kahoot!, a widely used and free real-time platform for formative educational assessment. Participants can compete for top scores in game-based quizzes, surveys, puzzles, and discussion forums. Kahoot! was previously found to motivate medical students to study, prioritize topics, and self-reflect on their learning [35]. The platform was then applied as a simple, low-cost method to teach histopathology and proper identification of benign and malignant tumors, including cutaneous neoplasms. Subsequently, quiz questions and answers were presented in random order, and the 36 students participating were given 30 seconds to answer each question. Ultimately, percentages of correct quiz answers increased after use of Kahoot! compared to classroom teaching, and there was a notable decrease in the time needed to correctly answer questions. Students evaluated their Kahoot! educational experience positively [22].

Skinquizition is another serious game using educational quizzes for formative assessment [23]. The game was developed especially for dermatology, as it allowed questions about diverse skin diseases, incorporated images, and could assess a large amount of applied knowledge in a short period of time. Assigned medical student laboratory groups were pitted against each other in a countdown-timed competition to correctly answer dermatology questions with an audience response system, popularly known as “clickers.” To incentivize active participation throughout the competition and even the odds between teams, teams were periodically offered the option to wager some or all of their points on the next question before seeing it, similar to a “Final Jeopardy” round. The gaming elements of a countdown timer, a wagering system, and prizes for winning teams made Skinquizition a thrilling learning experience for students. Afterwards, average class dermatology examination scores improved, along with student motivation and engagement.

Similar elements from *Jeopardy* have been leveraged to exciting effect at the American Academy of Dermatology annual meetings, where resident teams can compete in competitions related to dermatology and dermatopathology knowledge, trivia, and correct diagnoses of cases and images. Multiple fast-paced competition rounds and single elimination of contestants in front of a national conference audience have made the game show events perennial favorites among trainees. Prizes have included educational grants [25], gift cards [24], and “bragging rights.”

i-DERMIFY is an additional serious game learning tool developed to specifically harness dermatology’s visual and descriptive nature [26]. Teams of medical students drew action cards to either illustrate or describe skin conditions to each other, with hints provided when a difficult diagnosis was drawn. Each skin condition in the game was part of the British Association of Dermatologists’ required medical undergraduate curriculum. The game was iteratively developed with action research, where reflections from 28 student participants guided improvements to later games over time. Average assessment scores showed statistically significant positive increases (compared to knowledge gained from standard teaching, *P*<.005) after playing. Student confidence in describing, drawing, and recognizing skin diseases also grew. As with other studies of serious games, students responded positively and appreciated the learning techniques of visual representation and group work promoted by the game. In addition to building confidence and recognition during the limited time available for dermatology learning, i-DERMIFY also had the advantage of building the transferable clinical skills of accurately documenting skin conditions in medical records, and it allowed students to practice the concise verbal descriptions of skin lesions that are essential to communicating as future physicians. Thereafter, the game was incorporated into the regular medical school curriculum and made freely available for educational use.

Because serious educational games have been found to build confidence and reduce student stress and anxiety, the card-based board game Stud2yBuddy was specially designed to help medical students study dermatology for final examinations [27]. Learning objectives were mapped from a traditional British medical curriculum, and developers deliberately incorporated different learning styles into the game. Student attitude surveys reported increased confidence among students in the diagnosis, investigation, management, description, and recognition of dermatological conditions. However, no objective measures outside of Likert responses from these surveys of student confidence were assessed, though the 65 students participating did describe increased motivation and decreased stress after playing the game, as was originally expected.

Blending a serious game with a simulation in an “escape room” format also worked well for dermatology trainees, similar to the results of aforementioned studies in general medical education. Surveys of British medical students previously established that trainees have misconceptions about the field of dermatology, along with feelings of being inadequately prepared to manage skin conditions. It was speculated that these were possible barriers to engagement with dermatology as a specialty. After participating in a “zombie apocalypse”–themed escape room featuring a deadly skin disease outbreak, which consolidated previous lecture-based and clinical teaching, the majority of students reported a desire to experience more dermatology, and all enjoyed the session [28]. However, only 16 students were ultimately able to participate due to logistical and practical challenges with securing the physical venue for the escape room. Although further analysis with large-scale standardized studies is needed, our examination of available dermatology literature provides promising evidence for disrupting the paradigm of traditional classroom-based dermatology learning with both serious games and blended simulations.

### Gamification in Patient Dermatology Education

Successfully implemented gamification strategies in medical and dermatology education have also been applied to patient education, especially in regard to recognizing melanoma. Because most malignant melanomas are initially identified by the patients themselves or their partners, impactful melanoma education for the general public is essential [36]. Past efforts have focused on distributing pamphlets in physicians’ offices, a nonoptimal education format given that patients are increasingly web-centered and now receive most health care information from internet-based sources. Noninteractive websites may also be too passive for comprehensive patient understanding. An important consideration in the development of education materials is that patient education varies, and it often differs greatly from the training received by medical professionals. Although there are time constraints in patient counseling and necessity for individualization, given different personal needs, priorities, and resources, gamification approaches have still been effective for patients.

One example of a game-based intervention for nonmedical learners successfully taught features of malignant melanoma and alarming lesions. In a study of 271 adolescents, participants who were randomly selected to learn from a gamified six-round image matching program with built-in feedback such as “this lesion image shows irregular borders and uneven coloration, notice the irregular black spots on the left edge of this melanoma” performed significantly better than those receiving traditional “ABCDE” melanoma identification instruction [29]. Another study, developed by the Mayo Clinic, used a web-based pattern recognition game called Tapamole, which showed ABCDE melanoma examples (25 images of malignant melanoma and 60 of benign nevi). Volunteers were able to complete the game in <5 minutes, and they later demonstrated significantly greater sensitivity in recognizing malignant melanoma compared to their preintervention assessment performance. A follow-up Tapamole trial comparing the game, a traditional informational pamphlet, and no intervention showed similar melanoma detection sensitivity results between the game and pamphlet, but participants in the Tapamole game group reported the highest level of enjoyment [30]. Although many outcome measures reported by these studies were subjective (eg, motivation and enjoyment by the participants), these findings are vital to the development of future game-based learning programs, as patient willingness to participate in these programs will be key in moderating the long-term effects of patient education and driving persistence of engagement.

## Discussion

### Principal Findings

It is apparent that relatively few gamification studies specifically focused on dermatology currently exist in the literature, and of those studies, many are limited by small sample sizes or poor study design; for example, a control group where the gamification intervention was not administered was often lacking. Small numbers of participants and effect sizes as well as flaws in study design were detrimental to the quality of reported evidence. Our results corroborate recent systematic reviews of gamification interventions in general health care education, which have recognized that the extent of any positive impact is highly variable and nonuniformly measured [37] and largely of limited generalizable quality [3]. Additionally, the skills necessary to develop engaging games in dermatology (and health care education in general) may not directly overlap with the strengths of traditional medical educators; this would require more extensive collaboration with organizations outside of higher education and commitment of significant resources. The implementation of serious games and simulations can be both expensive and time-consuming. To that extent, some experts have suggested that gamification be considered part of an iterative process for directing learner engagement, rather than an end-product in itself [38]. Future investigations focusing on more robust, theory-driven methods should be encouraged. This evidence is complemented by generally positive attitudes regarding gamification by learners and educators; in one recent survey, 92% of residency program directors supported the use of games as an educational strategy [39], matching overwhelmingly positive responses from students [22].

### Limitations of This Narrative Review

Our narrative review is limited by its highly simplified search strategy, in which only a small subset of English-language peer-reviewed publications indexed in PubMed, Google Scholar, and ResearchGate were examined in detail. A secondary systematic review could be beneficial in extending the preliminary survey we have presented. Gamification is a relatively new approach to education, made possible by the increasing ubiquity of the internet and digital applications in daily life. Given the extreme pace of technology development, it follows that in-depth scientific studies of many gamified platforms may not yet be published; for example, a new iPhone and Android mobile game called “Top Derm: A Game for Dermatologists” is being increasingly advertised on Facebook and Doximity as of July 2021 [40]. Created by board-certified dermatologists, “Top Derm” allows users with an interest in dermatology to attempt challenges testing dermatology knowledge, gain experience points through answering diagnosis quizzes, and unlock achievements. The information presented in the game is based on verified medical knowledge resources, and the game may be eligible for CME credits in the near future. While the game may hold great potential for effective gamified dermatology learning, subsequent investigation of this and other applications is required before evidence-based recommendations can be established.

### Conclusion

Overall, previous studies indicate that gamification approaches—both serious games and simulations—provide a promising means of future dermatology-related education for students, professionals, and patients. The numerous advantages and reviews of gamification in medical education, however, are in stark contrast to the dearth of available gamification resources for dermatology-specific learning. Moreover, the moderate observed benefits for learner engagement and knowledge require further examination beyond pilot studies. Although there may be widespread support for gamification, our review of current literature provides a case for further investment in large-scale studies of gamified educational platforms, especially in dermatology, and more robust assessment of the true long-term impact of this learning enhancement on standardized outcomes.

## Abbreviations

CME: Continuing Medical Education
JAAD: Journal of the American Academy of Dermatology
JID: Journal of Investigative Dermatology
SIU: Société Internationale d’Urologie
